# CASP-8 −652 6N ins/del polymorphism and cancer risk: A literature-based systematic HuGE review and meta-analysis

**DOI:** 10.3892/etm.2012.661

**Published:** 2012-08-13

**Authors:** DA CHEN, TAO MA, XIAO-WEI LIU, ZHI LIU

**Affiliations:** Department of Emergency, First Affiliated Hospital, China Medical University, Shenyang, Liaoning, P.R. China

**Keywords:** apoptosis, caspase-8, polymorphism, cancer risk, meta-analysis

## Abstract

Caspase-8 (encoded by the CASP-8 gene) is crucial in generating cell death signals and eliminating potentially malignant cells. Genetic variation in CASP8 may affect susceptibility to cancer. The CASP-8 −652 6N ins/del (rs3834129) polymorphism has been previously reported to influence the progression to several cancers. However, the overall reported studies have shown inconsistent conclusions. In this human genome epidemiology (HuGE) review and meta-analysis, the aim was to identify the association between CASP-8 −652 6N ins/del polymorphism and cancer risk. According to the inclusion criteria, 19 case-control studies with a total of 23,172 cancer cases and 26,532 healthy controls were retrieved. Meta-analysis results showed that the del allele, del allele carrier and ins/del genotype of −652 6N ins/del in the CASP-8 gene were negatively associated with cancer risk (OR=0.91, 95% CI=0.84–0.98, P=0.01; OR=0.88, 95% CI=0.80–0.96, P=0.005; OR=0.91, 95% CI=0.85–0.98, P<0.001; respectively, while no significant correlation was observed between the del/del genotype of −652 6N ins/del and cancer risk (OR=0.89, 95% CI=0.79–1.01, P=0.08). In the subgroup analysis by ethnicity, the meta-analysis indicated that Caucasian populations harboring the del allele, del allele carriers and ins/del genotype had a lower cancer risk (OR=0.96, 95% CI=0.93–1.00, P=0.05; OR=0.86, 95% CI=0.75–1.00, P=0.05; OR=0.91, 95% CI=0.84–0.98, P=0.01; respectively). In addition, a negative association was found between the del allele of −652 6N ins/del in the CASP-8 gene and cancer risk in the Asian population (OR=0.89, 95% CI=0.83–0.97, P=0.005). In conclusion, this meta-analysis suggests that the del allele, del allele carrier and ins/del geno-type of the −652 6N ins/del polymorphism in the CASP-8 gene may be protective factors for cancer risk.

## Introduction

Cancer is a leading cause of death worldwide, with millions of individuals succumbing to various types of cancer annually (1). Therefore, it is of utmost importance to identify anticancer prevention and treatment strategies. According to epidemiology, cell apoptosis plays a role in the incidence of cancers. Apoptosis, also known as programmed cell death, is a fundamentally important biological process triggered by a variety of stimuli, including deprivation of growth/survival factors, exposure to cytotoxic drugs or DNA damaging agents, activation of death receptors and activity of cytotoxic cells, that is involved in controlling cell number and eliminating harmful or virus-infected cells to maintain cell homeostasis (2–4). The inappropriate process of apoptosis potentially results in various pathological disorders (5). The caspase family (cysteine and aspartic proteases) is mainly involved in the regulation of cell apoptosis (6), and has two major functions: caspase-1, −4, −5 and −11, as initiator caspases, are primarily involved in the processing and activation of pro-inflammatory cytokines, while caspase-2, −3, −6, −7, −8 and −9, as executor caspases, play a role in the execution phase of apoptosis (6,7). CASP activation has two dinstinct albeit converging pathways: the extrinsic or receptor-mediated pathway, and the intrinsic or mitochondrial pathway. These two pathways possess an independent group of initiator caspases despite using the same group of effector caspases (8–10). Caspase-8 (CASP-8) is essential for the extrinsic cell death pathways initiated by the TNF family members with the formation of the death-inducing signaling complex (11).

Single-nucleotide polymorphisms (SNPs) are the most common form of human genetic variation, leading to susceptibility to cancer. Findings of previous studies showed that some variants in CASP-8 gene are associated with susceptibility to various human cancers (12,13). A case-control study in a Chinese population found that CASP-8 −652 6N del/del genotypes showed a multiplicative joint effect with FasL and Fas in attenuating susceptibility to pancreatic cancer (14). However, relevant studies on −652 6N del in CASP-8 are inconclusive and inconsistent. Therefore, a human genome epidemiology (HuGE) review and meta-analysis were conducted, including the most recent and relevant articles in order to identify statistical evidence of the association between the CASP-8 −652 6N ins/del polymorphism and cancer risk that have been investigated.

## Materials and methods

### Literature search

An extensive electronic search of the PubMed, Cochrane Library, Embase, Web of Science, SpringerLink, CNKI and CBM databases was performed to identify relevant studies available up to May 1, 2012. The search terms used included [‘caspase-8’, ‘CASP-8’ or ‘Caspase 8’ (Mesh)] and [‘SNPs’, ‘SNP’ or ‘polymorphism, genetic’ (Mesh)] and [‘cancer’, ‘tumor’ or ‘Neoplasms’ (Mesh)]. The references in the eligible studies or textbooks were also reviewed to check through manual searches to find other potentially eligible studies.

### Inclusion and exclusion criteria

The included studies had to meet the following criteria: i) case-control study focused on the associations between CASP-8 −652 6N ins/del polymorphism and cancer risk; ii) all patients diagnosed with a malignant tumor confirmed by pathological examination of the surgical specimen; iii) the frequencies of alleles or geno-types in case and control groups could be extracted; iv) the publication was in English or Chinese. Studies were excluded when they were: i) not case-control studies about CASP-8 −652 6N ins/del polymorphism and cancer risk; ii) based on incomplete data; iii) useless or overlapping data were reported; iv) meta-analyses, letters, reviews or editorial articles.

### Data extraction

Using a standardized form, data from published studies were extracted independently by two reviewers to populate the necessary information. The information extracted from each of the articles included: first author, year of publication, country, language, ethnicity, study design, source of cases and controls, number of cases and controls, mean age, sample, cancer type, genotype method, allele and genotype frequency, and evidence of Hardy-Weinberg equilibrium (HWE) in controls. In case of conflicting evaluations, an agreement was reached following a discussion with a third reviewer.

### Quality assessment of included studies

Two reviewers independently assessed the quality of papers according to modified STROBE quality score systems (15,16). Forty assessment items associated with the quality appraisal were used in this meta-analysis, scores ranging from 0 to 40. Scores of 0–20, 20–30 and 30–40 were defined as low, moderate and high quality, respectively. Disagreement was resolved by discussion.

### Statistical analysis

The odds ratio (OR) and 95% confidence interval (95% CI) were calculated using Review Manager Version 5.1.6 (provided by the Cochrane Collaboration, available at: http://ims.cochrane.org/revman/download) and STATA Version 12.0 (Stata Corp., College Station, TX, USA) software. Between-study variations and heterogeneities were estimated using Cochran’s Q-statistic (17,18) (P≤0.05 was considered to be a manifestation of statistically significant heterogeneity). The effect of heterogeneity, ranging from 0 to 100% and representing the proportion of inter-study variability that can be contributed to heterogeneity rather than to chance, was quantified using the I2 test. When a significant Q-test (P≤0.05) or I^2^>50% indicated that heterogeneity among studies existed, the random-effects model was employed for the meta-analysis. Otherwise, the fixed-effects model was used. To establish the effect of heterogeneity on conclusions of the meta-analyses, a subgroup analysis was carried out. We also tested whether genotype frequencies of controls were in HWE using the χ^2^ test. Funnel plots are often used to detect publication bias. However, due to its limitations caused by varied sample sizes and subjective reviews, Egger’s linear regression test, which measures the funnel plot’s asymmetry using a natural logarithmic scale of OR, was used to evaluate the publication bias (19). When the P-value is <0.1, publication bias is considered significant. All the P-values were two-sided. To ensure the reliability and accuracy of the results, two reviewers populated the data in the statistical software programs independently and obtained identical results.

## Results

### Characteristics of included studies

**Figure 1:**
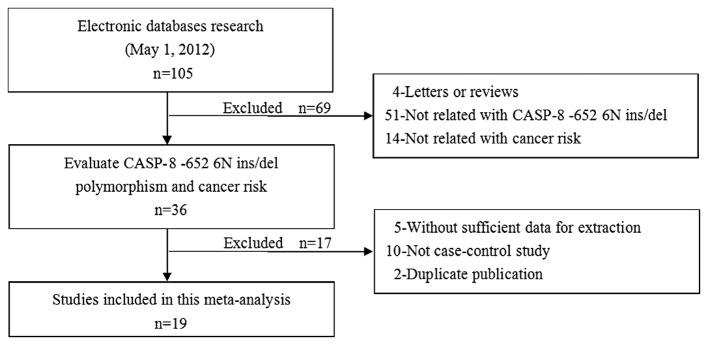
Flow chart shows study selection procedure. Nineteen case-control studies were included in this meta-analysis.

Subsequent to the initial screening a total of 105 relevant publications were identified. Nineteen studies (20–37) appeared to have met the inclusion criteria and were subjected to further examination. The flow chart of study selection is shown in Fig. 1. In the pooled analysis, a total of 23,172 cancer cases and 26,532 healthy controls from 19 studies were included and addressed. The publication year of involved studies ranged from 2006 to 2011. Twelve of these studies were conducted in Asian populations, 6 in Caucasian populations and 1 in African populations. The HWE test was performed on the genotype distribution of the controls in all the included studies, 2 of these studies were out of HWE (34,37) and the remaining studies showed to be in HWE (P>0.05). Quality scores of included studies were >20 (moderate-high quality). The characteristics and methodological quality of the included studies are shown in Table I. The genotype distribution of the CASP-8 −652 6N ins/del polymorphism in the case and control groups is shown in Table II.

### Main results and subgroup analysis

**Figure 2:**
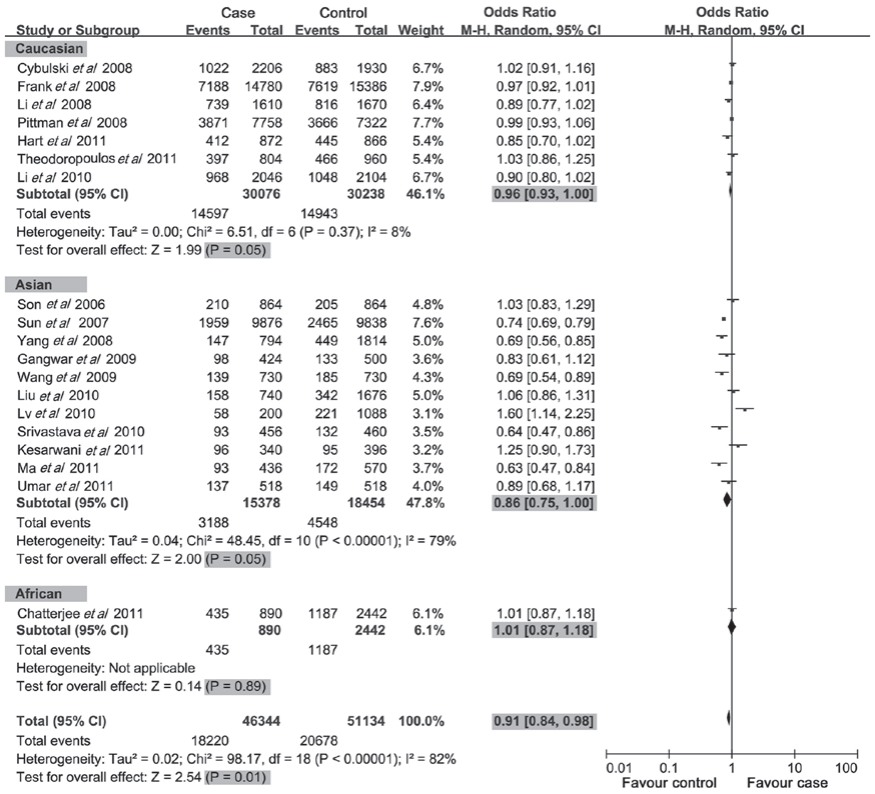
Associations between del allele of the −652 6N ins/del polymorphism and cancer risk.

**Figure 3:**
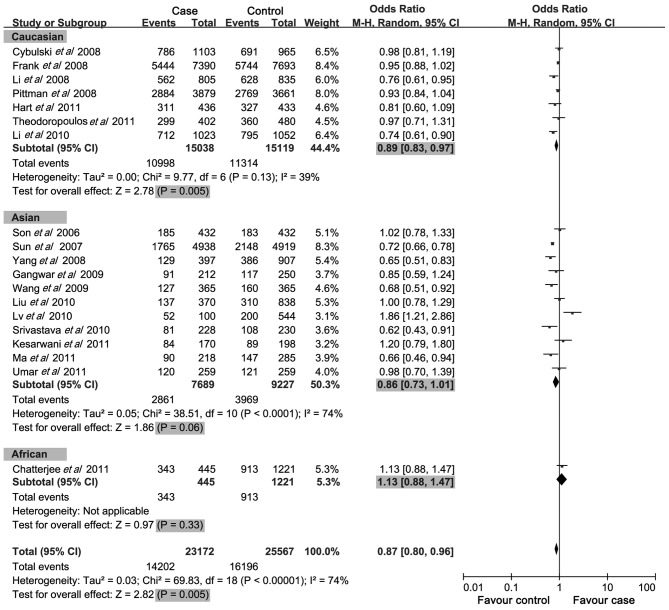
Associations between the del allele carrier of the −652 6N ins/del polymorphism and cancer risk.

**Figure 4:**
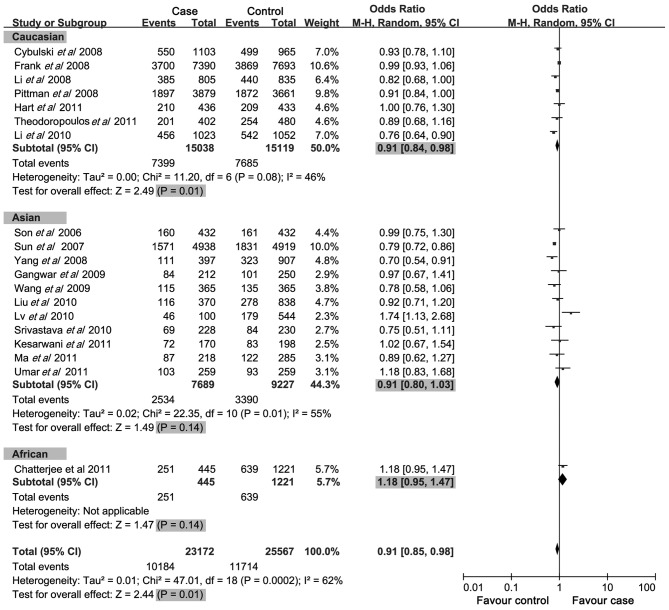
Associations between the ins/del genotype of the −652 6N ins/del polymorphism and cancer risk.

A summary of the meta-analysis findings of the association between CASP-8 −652 6N ins/del polymorphism and cancer risk is provided in Table III. The meta-analysis results showed that the del allele, del allele carrier and ins/del genotypes of −652 6N ins/del in CASP-8 gene were negatively associated with cancer risk (OR=0.91, 95% CI=0.84–0.98, P=0.01; OR=0.88, 95% CI=0.80–0.96, P=0.005; OR=0.91, 95% CI=0.85–0.98, P<0.001; respectively) (Figs. 2–4), while no significant correlation was observed between the del/del genotypes of −652 6N ins/del and cancer risk (OR=0.89, 95% CI=0.79–1.01, P=0.08). In the subgroup analysis by ethnicity, we found that the del allele of −652 6N ins/del was a protective factor for cancer risk in the Caucasian and Asian populations (OR=0.96, 95% CI=0.93–1.00, P=0.05; OR=0.86, 95% CI=0.75–1.00, P=0.05; respectively), although not in the African population (OR=1.01, 95% CI=0.87–1.18, P=0.891). For the del allele carrier of −652 6N ins/del polymorphism, negative associations with cancer risk were found in the Caucasian population (OR=0.89, 95% CI=0.83–0.97, P=0.005), but not in the Asian and African populations (OR=0.86, 95% CI=0.73–1.01, P=0.06; OR=1.13, 95% CI=0.88–1.47, P=0.33; respectively). Notably, no associations were found between the del/del genotype (variant homozygote) of the −652 6N ins/del polymorphism and cancer risk in the three populations (OR=0.89, 95% CI=0.79–1.10, P=0.08). However, with regards to the ins/del genotype (heterozygote) of the −652 6N ins/del polymorphism, protective associations with cancer risk were found in the Caucasian population (OR=0.91, 95% CI=0.84–0.98, P=0.01), whereas no correlation was found in the Asian and African populations (OR=0.91, 95% CI=0.80–1.03, P=0.14; OR=1.18, 95% CI=0.95–1.47, P=0.14; respectively).

### Publication bias

**Figure 5:**
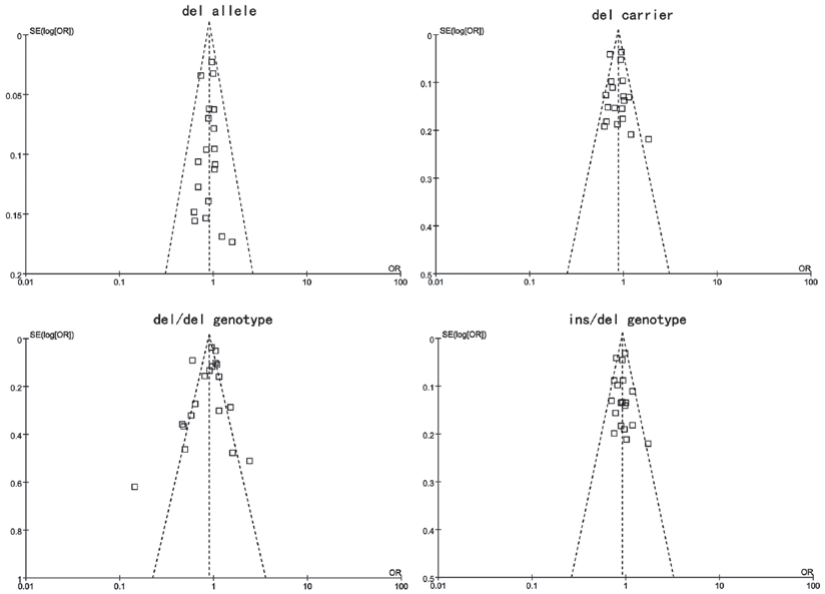
Begger’s funnel plot of publication bias.

Publication bias of the literature was accessed by Begger’s funnel plot and Egger’s linear regression test. Egger’s linear regression test was used to measure the asymmetry of the funnel plot. The graphical funnel plots of included studies appeared to be symmetrical (Fig. 5). Egger’s test also showed that there was no statistical significance for all evaluations of publication bias (all P>0.05). Findings of Egger’s publication bias test are shown in Table IV.

## Discussion

CASP-8, located on chromosome 2q33–q34, encoded by the CASP-8 gene, is a caspase protein that plays a key role in the execution-phase of cell apoptosis (28). When induced by Fas and various apoptotic stimuli, this protein is involved in apoptosis (29). Caspase-8 is known to activate during death receptor-initiated apoptosis, inducing apoptosis and maintaining immune homeostasis and immune surveillance, while the single genetic variants in CASP-8 and their function in human cancer susceptibility remain to be elucidated (21). The −652 6N ins/del (rs3834129), a common SNP in the CASP-8 gene, is strongly associated with the CASP-8 expression. Investigators have reported a correlation between the −652 6N ins/del polymorphism and susceptibility to various types of cancer. Sun *et al* observed that the CASP-8 −652 6N ins/del allele was associated with a reduced risk of developing different types of human cancer, including lung, esophageal, colorectal, cervical and breast cancer, as well as gastric cancer, indicating that this variant allele may confer protection against multiple cancers (21). Frank *et al* showed that the CASP-8 −652 6N ins/del variant has no significant effect on breast cancer risk in Europeans (23). In their study, Li *et al* observed that the CASP-8 −652 6N ins/del variant genotypes (ins/del, ins/del+del/del) were associated with significantly lower cutaneous melanoma risk than were the ins/ins genotypes (24). In our study, we examined the association of the −652 6N ins/del polymorphism in the CASP-8 gene with the risk for cancer by meta-analysis. A negative association was observed between the del allele, del allele carrier and ins/del genotype of the −652 6N ins/del polymorphism in CASP-8 gene and cancer risk. In the stratified analysis by ethnicity, Caucasians who harbored the ins/del genotypes or del allele or del allele carrier were found to exhibit a significantly lower risk for cancer. In addition, a negative association was also found between the del allele of −652 6N ins/del in CASP-8 gene and cancer risk in the Asian population.

Limitations of this study should be acknowledged. First, although the funnel plot and Egger’s test did not show any publication bias, selection bias may have occurred because only studies published in English or Chinese were included. Second, the control subjects of the present study might not be representative of the general population, necessitating well-designed population-based studies with large sample sizes and detailed exposure information to validate our findings. Third, there was significant between-study heterogeneity from studies of the −652 6N ins/del polymorphism, while the geno-type distribution also showed deviation from HWE in some studies. Fourth, our meta-analysis was based on unadjusted OR estimates as not all published studies presented adjusted ORs, or when they did, the ORs were not adjusted by the same potential confounders, such as age, gender, ethnicity and exposures. In addition, our analysis did not consider the possibility of gene-gene or SNP-SNP interactions or the possibility of linkage disequilibrium between polymorphisms. Therefore, our conclusions should be interpreted with caution.

In conclusion, findings of this study have shown a common insertion-deletion variation in the promoter region of the CASP-8 gene as a low penetrance susceptibility locus for certain common types of human cancers. The del allele, del allele carrier and ins/del genotype of the −652 6N ins/del polymorphism in CASP-8 gene may serve as protective factors for cancer risk. However, these findings should be validated by large-scale, prospective studies investigating more diverse ethnic groups and more detailed environmental exposure data.

## Figures and Tables

**Table I:** Characteristics of individual studies in this meta-analysis.

Authors (Refs.)	Year	Country	Case no.		Sample	Genotype method	Cancer type	Quality scores
			AS	Control				
Son *et al* (20)	2006	Korea	432	432	Blood	PCR-RFLP	Lung cancer	27
Sun *et al* (21)	2007	China	4995	4972	Blood	PCR-RFLP	Mixed cancer	23
Cybulski *et al* (22)	2008	Poland	618	531	Blood	AS-PCR	Mixed cancer	20
Frank *et al* (23)	2008	Germany	7753	7921	Blood	FFA	Breast cancer	23
Li *et al* (24)	2008	China	805	835	Blood	PCR-RFLP	Melanoma	26
Pittman *et al* (25)	2008	UK	4016	3749	Blood	AS-PCR	Colorectal cancer	21
Yang *et al* (14)	2008	China	397	907	Blood	PCR-RFLP	Pancreatic cancer	24
Gangwar *et al* (26)	2009	India	212	250	Blood	PCR-RFLP	Bladder cancer	29
Wang *et al* (27)	2009	China	365	368	Blood	PCR-RFLP	Bladder cancer	26
Li *et al* (28)	2010	USA	1023	1052	Blood	PCR-RFLP	Head and neck cancer	26
Liu *et al* (29)	2010	China	373	838	Blood	PCR-RFLP	Colorectal cancer	25
Lv *et al* (30)	2010	China	100	544	Blood	TaqMan	Lymphoma	26
Srivastava *et al* (31)	2010	India	230	230	Blood	PCR-RFLP	Gallbladder cancer	24
Chatterjee *et al* (32)	2011	South Africa	445	1221	Blood	PCR-RFLP	Cervical cancer	18
Hart *et al* (33)	2011	Norway	442	440	Blood/tissue	TaqMan	Lung cancer	20
Kesarwani *et al* (34)	2011	India	175	198	Blood	PCR-RFLP	Prostate cancer	24
Ma *et al* (35)	2011	China	218	285	Blood	Mass-Array	Ovarian cancer	18
Theodoropoulos *et al* (36)	2011	Greece	402	480	Blood	PCR-RFLP	Colorectal cancer	18
Umar *et al* (37)	2011	India	259	259	Blood	PCR-RFLP	Esophageal cancer	20

PCR, polymerase chain reaction; RFLP, restriction fragment length polymorphism; AS, allele specific; FFA, Fluorescent fragment analysis.

**Table II:** The genotype distribution of CASP-8 −652 6N polymorphism in case and control groups.

Authors (Refs.)	Case						Control						HWE test		
	Total	ins	del	ins/ins	ins/del	del/del	Total	ins	del	ins/ins	ins/del	del/del	χ^2^	P-value	Test
Son *et al* (20)	432	654	210	247	160	25	432	659	205	249	161	22	0.38	0.54	HWE
Sun *et al* (21)	4938	7917	1959	3173	1571	194	4919	7373	2465	2771	1831	317	0.39	0.53	HWE
Cybulski *et al* (22)	1103	1184	1022	317	550	236	965	1047	883	274	499	192	1.68	0.20	HWE
Frank *et al* (23)	7390	7592	7188	1946	3700	1744	7693	7767	7619	1949	3869	1875	0.27	0.60	HWE
Li *et al* (24)	805	871	739	243	385	177	835	854	816	207	440	188	2.47	0.12	HWE
Pittman *et al* (25)	3879	3887	3871	995	1897	987	3661	3656	3666	892	1872	897	1.88	0.17	HWE
Yang *et al* (14)	397	647	147	268	111	18	907	1365	449	521	323	63	1.76	0.19	HWE
Gangwar *et al* (26)	212	326	98	121	84	7	250	367	133	133	101	16	0.30	0.58	HWE
Wang *et al* (27)	365	591	139	238	115	12	365	545	185	205	135	25	0.19	0.67	HWE
Li *et al* (28)	1023	1078	968	311	456	256	1052	1056	1048	257	542	253	1.54	0.21	HWE
Liu *et al* (29)	370	582	158	233	116	21	838	1334	342	528	278	32	0.38	0.54	HWE
Lv *et al* (30)	100	142	58	48	46	6	544	867	221	344	179	21	0.15	0.70	HWE
Srivastava *et al* (31)	228	363	93	147	69	12	230	328	132	122	84	24	2.66	0.10	HWE
Chatterjee *et al* (32)	445	455	435	102	251	92	1221	1255	1187	308	639	274	2.75	0.10	HWE
Hart *et al* (33)	436	460	412	125	210	101	433	421	445	106	209	118	0.50	0.48	HWE
Kesarwani *et al* (34)	170	244	96	86	72	12	198	301	95	109	83	6	4.42	0.04	Non-HWE
Ma *et al* (35)	218	343	93	128	87	3	285	398	172	138	122	25	0.07	0.79	HWE
Theodoropoulos *et al* (36)	402	407	397	103	201	98	480	494	466	120	254	106	1.68	0.19	HWE
Umar *et al* (37)	259	381	137	139	103	17	259	369	149	138	93	28	3.97	0.05	Non-HWE

CASP-8, caspase-8; HWE, Hardy-Weinberg equilibrium.

**Table III:** Meta-analysis of the association between the −652 6N ins>del polymorphism in CASP-8 and cancer risk.

Subgroup	Case no./N	Control no./N	OR (95% CI)	P-value	Effect model
del allele	18220/46344	20678/51134	0.91 (0.84–0.98)	0.01	Random
Caucasian	14597/30076	14943/30238	0.96 (0.93–1.00)	0.05	
Asian	3188/15378	4548/18454	0.86 (0.75–1.00)	0.05	
African	435/890	1187/2442	1.01 (0.87–1.18)	0.89	
del allele carrier	14202/23172	16196/25567	0.87 (0.80–0.96)	0.005	Random
Caucasian	10998/15038	11314/15119	0.89 (0.83–0.97)	0.005	
Asian	2861/7689	3969/9227	0.86 (0.73–1.01)	0.06	
African	343/445	913/1221	1.13 (0.88–1.47)	0.33	
del/del	4018/23172	4482/25567	0.89 (0.79–1.01)	0.08	Random
Caucasian	3599/15308	3629/15119	1.00 (0.95–1.05)	0.90	
Asian	327/7689	579/9227	0.73 (0.53–1.01)	0.06	
African	92/445	274/1221	0.90 (0.69–1.18)	0.44	
ins/del	10184/23172	11741/25567	0.91 (0.85–0.98)	<0.001	Random
Caucasian	7399/125038	7685/15119	0.91 (0.84–0.98)	0.01	
Asian	2534/7689	3390/9227	0.91 (0.80–1.03)	0.14	
African	251/445	639/1221	1.18 (0.95–1.47)	0.14	

OR, odds ratio; 95% CI, 95% confidence interval.

**Table IV:** Evaluation of publication bias by Egger’s linear regression test.

SNP	Coefficient	SE	t	P-value	95% CI
del allele	−0.298	0.932	−0.320	0.753	(−2.265, 1.669)
del carrier	0.375	0.834	0.450	0.658	(−1.384, 2.135)
del/del genotype	−0.745	0.645	−1.160	0.264	(−2.105, 0.615)
ins/del genotype	0.192	0.664	0.290	0.776	(−1.208, 1.592)

SE, standard error; 95% CI, 95% confidence interval.
